# Creating a Powerful Platform to Explore Health in a Correctional Population: A Record Linkage Study

**DOI:** 10.1371/journal.pone.0161173

**Published:** 2016-08-17

**Authors:** Kathryn E. McIsaac, Shanna Farrell MacDonald, Nelson Chong, Andrea Moser, Rahim Moineddin, Angela Colantonio, Avery Nathens, Flora I. Matheson

**Affiliations:** 1 Dalla Lana School of Public Health, University of Toronto, Toronto, Ontario, Canada; 2 Centre for Urban Health Solutions, St. Michael’s Hospital, Toronto, Ontario, Canada; 3 Correctional Service Canada, Ottawa, Ontario, Canada; 4 Institute for Clinical Evaluative Sciences, Sunnybrook Hospital, Toronto, Ontario, Canada; 5 Department of Family and Community Medicine, University of Toronto, Toronto, Ontario, Canada; 6 Rehabilitation Sciences Institute, University of Toronto, Toronto, Ontario, Canada; 7 Department of Occupational Science and Occupational Therapy, University of Toronto, Toronto, Ontario, Canada; 8 Department of Surgery, University of Toronto, Toronto, Ontario, Canada; University of Rochester, UNITED STATES

## Abstract

We used record linkage to create a data repository of health information of persons who were federally incarcerated in Ontario and Canada. We obtained records from 56,867 adults who were federally incarcerated between January 1, 1998 and December 31, 2011 from the Correctional Service of Canada; 15,248 records belonged to individuals residing in Ontario, Canada. We linked these records to the Registered Persons Database (RPDB) which contained records from 18,116,996 individuals eligible for health care in Ontario. Out of 56,867 OMS records, 22,844 (40.2%) were linked to the RPDB. Looking only at those incarcerated in Ontario, 98%, (14 953 of 15248) records were linked to RPDB. Most records of persons in Ontario-based facilities were linked deterministically. Linkage rates were lower for women, minority groups, and substance users. In conclusion, record linkage enabled the creation of a valuable data repository: there are no electronic medical records for correctional populations in Canada, making it more difficult to profile their health.

## Introduction

An estimated 37,000 individuals are incarcerated in Canada on any given day and these individuals tend to be disproportionately burdened by poor health, including high rates of infectious disease, substance use, and mental illness[1–6]. Most data on the health of persons experiencing incarceration is based on cross-sectional surveys collected while in custody; there is little longitudinal research on the health of incarcerated persons outside of correctional facilities either prior to or post incarceration[7–9]. The one exception is mortality: several studies report a high risk of death immediately following custodial release[10, 11].

There are increasing calls to enrich our understanding of the health of persons who experience incarceration[12–15]. Such individuals typically draw from the most vulnerable and marginalized groups, groups that are less likely to access health care. Incarceration may improve health, providing an opportunity to diagnose and manage illness in traditionally under-serviced populations. On the other hand, it has been argued that incarceration may engender illness through environments (e.g. close person to person contact) and behaviours (e.g. risky sexual activities) that may lead to health implications. In order to truly understand if and how incarceration impacts health, longitudinal data are needed. Currently, there are no existing Canadian datasets that capture the health of federally incarcerated persons pre and post custody.

Record linkage could be a cost-effective strategy to generate data on the health of persons experiencing incarceration. Linkage studies capitalize on data collected for other purposes and the indicators in the resultant repositories enable research that would not have been possible from any data source alone[16]. Linkage studies can increase capacity to conduct research in vulnerable populations and such an approach has been used to examine the mortality of persons incarcerated post-release[17].

We performed a record linkage study, joining together Canadian federal correctional data and administrative health data. The resultant database is powerful and unique: one of only a few population-based, longitudinal datasets to capture a wide range of health and correctional indicators from incarcerated individuals in the world[17]. The present study describes the record linkage and examines the characteristics of individuals whose correctional records were more likely to be matched to administrative health data as well as the characteristics of those linked deterministically (i.e. exact matches).

## Materials and Methods

### Database Descriptions

Correctional data were obtained from the Correctional Service of Canada (CSC), the federal government agency responsible for administering sentences of two years or more, for adults aged 18 years or older. We extracted correctional data from the Offender Management System (OMS), a computerized record system that tracks information from admission until warrant expiry. The OMS contains: demographic characteristics; alternative names (i.e. aliases); criminal history; sentencing information (e.g. length); and behaviour while incarcerated (e.g. violent acts). These data elements are completed by institutional personnel. The OMS also contains information on risk factors that could have contributed to incarceration (e.g. substance use history, criminal history) used to inform programming needs as prescribed by the risk-need-responsivity model[18]. This model posits that rehabilitation will be more successful if treatment matches criminal needs[19]. Risk factor data are collected by self-report using validated instruments (e.g. Drug Abuse Screening Test)[20] and an overall criminal risk rating is calculated by CSC staff based on response patterns. The OMS will have data on all persons under federal custody.

Health data were obtained from the Institute for Clinical Evaluative Sciences (ICES), an autonomous not-for profit agency that holds administrative health data (e.g. physician billings, emergency room visits) for services provided in Ontario, Canada. In the present study, we linked the OMS to the Registered Persons Database (RPDB), the master list of anyone who is, or who has ever been, eligible for health care in Ontario. The RPDB contains basic demographics–surname and first name, date of birth, sex, postal code—as well as a unique health card identifier, enabling linkage with other health utilization data. It is maintained by Ontario’s Ministry of Health and Long Term Care. Because Canada has universal health care coverage, all Canadian citizens and permanent residents living in Ontario should be included in the RPDB[21].

### Inclusion Criteria

Our OMS database contained records from 56,867 adults admitted to a federal correctional facility between January 1, 1998 and December 31, 2011. The database excluded those incarcerated before January 1, 1998; who served only a provincial or territorial sentence; or who had their record suspended (i.e. pardoned). The RPDB contained records from 18,116,996 individuals eligible for health care in Ontario between April 1, 1990 and July 2014, the latter date being when the linkage was performed.

### Data Sharing Agreement

Prior to data linkage, two privacy impact assessments were conducted and a data sharing agreement was struck between ICES and CSC in July 2013. This legal agreement outlined the terms and conditions of data sharing, including security measures, approved uses of data, and destruction of information. Personal identifiers were available to three data covenantors for the purposes of record linkage and these individuals have approved access to such information by the Ontario Information and Privacy Commissioner. All data covenantors underwent a security background check by CSC. Once completed, covenantors were granted “reliability status”, or ability to access the data. This study was approved by the institutional review board at Sunnybrook Health Sciences Centre, Toronto, Canada. We also received approval from the Research Ethics Boards of St. Michael’s Hospital and the University of Toronto.

### Database preparation

Aliases are common in correctional populations and failing to account for these may decrease the sensitivity of linkage[22]. To prepare the data for linkage, one record was added to the OMS database for each unique surname/alias. These records also included the first name, gender, date of birth, and postal code.

### Data linkage

All linkage was performed using the Automatch software[23]. We used surname, given name, sex, date of birth, and residential postal code to link OMS records to the RPDB. These data elements are assumed to be accurate in both databases and contain no missing fields. We linked records using both deterministic and probabilistic approaches. Deterministic linkage requires perfect agreement on specified data fields; these data fields should be sufficiently unique so that only one person can be ascribed to the record. Probabilistic linkage is used when linking data fields may not be unique. In the face of such complexity, statistical probabilities, called linkage weights, are generated reflecting the likelihood two records are a true match[23].

In the present study, linkage occurred in five different passes: records linked on the most recent pass were removed from the linkage pool. The first pass used deterministic linkage, requiring an exact match on surname, given name, date of birth and sex. This combination of data elements produced a *correct* linkage rate of over 98% in another Canadian study[24]. The second through fifth passes used probabilistic linkage, requiring agreement on fewer data fields or strings of data (e.g. first three letters of a surname). Match requirements for each pass are found in Table 1. At each probabilistic linkage pass, linkage weights were generated by the software. Weights were based on the *m* and *u* probabilities which are the conditional probability that a field agrees, given the pair is a true match and the conditional probability a field agrees, given the pair is a true mismatch, respectively. A total linkage weight is calculated from these *m* and *u* probabilities[23]. Linked pairs with weights falling above pre-determined thresholds were considered automatic matches whereas pairs with weights falling below thresholds were considered non-matches. Linked pairs with weights falling within the threshold range were considered possible matches and were manually reviewed by two data covenantors. Postal codes were considered during the manual review. Rules were created *a priori* to determine if manually reviewed pairs would be accepted or rejected as matches and are listed in S1 File.

**Table 1:** Data fields used to link Correctional Service Canada data to the Registered Persons Database, by pass number

Pass	Data elements
1	Surname + given name + date of birth + sex
2	First 3 initials of surname + given name initial + date of birth + sex
3	Date of birth + sex
4	First 3 initials of surance + given name initial + birth year + sex
5	Surname + sex

Because of the matching algorithm and decision rules, it was possible that multiple unique correctional records could be matched to the same health record on the same pass. When this occurred, we considered the true match to be the record with the highest linkage weight, i.e. the record that was statistically more likely to be a true match.

### Study outcome

We examined the proportion of OMS records that were linked to the RPDB (the linkage rate). We acknowledge the percent of records linked is a proportion and not a true rate and use this terminology to be consistent with terminology used elsewhere[16, 24]. We then subdivided linked individuals by probabilistic or deterministic linkage.

### Indicators of interest

We examined linkage rates by demographic characteristics and criminal risk and needs as recorded in the OMS. Demographic characteristics were age, sex, race, marital status and number of aliases. Criminal needs were substance use and dependence history, measured using the Alcohol Dependence Scale (ADS)[25], the Problems Related to Drinking Scale (PRD), based on the Michigan Alcohol Screening Test (MAST)[26], and the Drug Abuse Screening Test (DAST)[20]. These scales have been validated in correctional populations[27, 28]. Over the study period, scales were administered to individuals with suspected substance dependence (prior to 2009) or everyone admitted to the federal correctional facility (2009-onward). We also examined linkage rates by Static Factor Rating, henceforth referred to as criminal history risk score. Risk scores are designed to predict risk of re-offending post-release, based on unmodifiable factors, e.g. criminal history[29]. These measures were developed by CSC as part of the Offender Intake Assessment process and have been in use since 1994[28, 30].

### Data analysis

We calculated the linkage rate, overall and by select characteristics of interest. The majority of analyses focused on a subsample of individuals admitted to a federal institution in Ontario (n = 15,248). Admission region is highly correlated with the province where an individual committed their crimes and were sentenced. Because we only have access to Ontario health data, this approach should increase the linkage accuracy.

We used logistic regression to calculate the odds of being a) linked compared with not linked and b) linked deterministically compared with probabilistically. These datasets were analyzed at the Institute for Clinical Evaluative Sciences (ICES).

## Results

Out of 56,867 OMS records, 22,844 (40.2%) were linked to the RPDB and 34,023 (59.8%) were not. After assuming duplicate matches with the lowest linkage weights were false positives (n = 29), the linkage rate was 40.1% (22,815 of 56,867).

Looking exclusively at individuals admitted to an Ontario-based facility, the linkage rate increased to 98% (14,953 of 15,248): only 295 individuals were not linked (2%). Outside of Ontario, 18.9% of records were linked (7,862 of 41,619). The overwhelming majority of matched records were linked deterministically (i.e. on the first pass): 78.6% of all *matched* records (17,941 of 22,815) and 85.6% of *all* Ontario-based records were linked deterministically (13,057 of 15,248). Within Ontario, 8.1% (n = 1235), 3.6% (n = 549), 0.6% (n = 100), and <0.1% (n = 12) of *all* records were linked on the second through fifth passes, respectively.

The mean number of OMS records per unique person (i.e. aliases) was 2.56 (SD = 2.00) and 3.23 (SD = 2.83) for men and women incarcerated in Ontario. Aliases were less common among individuals incarcerated outside of Ontario: the average number of names was 2.20 (SD = 2.26) for men and 1.98 (SD = 2.15) for women. The maximum number of surnames was 53.

Table 2 shows the distribution of matched records, by linkage rate, in Ontario. We found that women, minority populations, and individuals with more severe alcohol use were less likely to be linked than their respective comparator (column 6). We found that individuals with a greater number of aliases or a higher risk of reoffending were more likely to be linked.

**Table 2:** Characteristics of federally incarcerated persons in the province of Ontario (1998–2011), by linkage outcome (N = 15,248).

Characteristics		Linkage typeN, %			Odds RatioOR, 95% CI	
	n	Deterministic N = 13,057	Probabilistic N = 1896	No Linkage N = 295	Linked vs. Not Linked N = 15,248	Deterministic vs. Probabilistic N = 14,953
Age (years)						
<25	3178	2713 (85.4)	405 (12.7)	60 (1.9)	0.96 (0.69, 1.32)	0.95 (0.82, 1.07)
25–34	5259	4529 (86.1)	635 (12.1)	95 (1.8)	1.00	1.00
35–44	3954	3369 (85.2)	507 (12.8)	78 (2.0)	0.91 (0.68, 1.24)	0.93 (0.82, 1.06)
45–54	1997	1711 (85.7)	246 (12.3)	40 (2.0)	0.90 (0.62, 1.31)	0.98 (0.83, 1.14)
55+	860	735 (85.4)	103 (12.0)	22 (2.6)	0.70 (0.44, 1.12)	1.00 (0.80, 1.25)
Sex						
Men	14221	12180 (85.6)	1777 (12.5)	264 (1.9)	1.00	1.00
Women	1027	877 (85.4)	119 (11.6)	31 (3.0)	**0.61 (0.42, 0.89)**	1.08 (0.88, 1.31)
Race						
White	9450	8250 (87.3)	1081 (11.4)	119 (1.3)	1.00	1.00
Aboriginal	1494	1234 (82.6)	168 (11.2)	92 (6.2)	**0.19 (0.15, 0.26)**	0.96 (0.81, 1.14)
Black	2609	2221 (85.1)	339 (13.0)	49 (1.9)	**0.67 (0.48, 0.93)**	**0.86 (0.75, 0.98)**
Other ^a^	1695	1352 (79.8)	308 (18.2)	35 (2.1)	**0.60 (0.41, 0.88)**	**0.58 (0.50, 0.66)**
Marital status						
Married, common law	6266	5360 (85.5)	776 (12.4)	130 (2.1)	1.00	1.00
Other ^b^	8982	7697 (85.7)	1120 (12.5)	165 (1.8)	1.13 (0.90, 1.43)	1.00 (0.90, 1.10)
Number of aliases						
0	5063	4427 (87.4)	506 (10.0)	130 (2.6)	1.00	1.00
1–2	6924	5977 (86.3)	820 (11.8)	127 (1.8)	**1.41 (1.10, 1.81)**	**0.83 (0.74, 0.94)**
3+	3261	2653 (81.4)	570 (17.5)	38 (1.2)	**2.24 (1.55, 3.22)**	**0.53 (0.47, 0.60)**
Alcohol Problems ^c^						
None	9514	8198 (86.2)	1166 (12.3)	150 (1.6)	1.00	1.00^h^
Some	1752	1508 (86.1)	213 (12.2)	31 (1.8)	0.89 (0.60, 1.31)	1.01 (0.86, 1.18)
Quite a few or a lot	2436	2087 (85.6)	278 (11.4)	51 (2.9)	**0.53 (0.40, 0.71)**	1.07 (0.93, 1.23)
Missing	1546	1264 (81.8)	239 (15.5)	43 (2.8)	**0.56 (0.40,0.79)**	**0.75 (0.65, 0.88)**
Alcohol Dependence ^d^						
None	8188	7038 (86.0)	1019 (12.4)	131 (1.6)	1.00	1.00 ^h^
Low to Moderate	4849	4195 (86.5)	553 (11.4)	102 (2.1)	**0.76 (0.58, 0.98)**	1.10 (0.98, 1.23)
Substantial or Severe	665	561 (84.4)	85 (12.8)	19 (2.9)	**0.55 (0.34, 0.90)**	0.96 (0.75, 1.21)
Missing	1546	1264 (81.8)	239 (15.5)	43 (2.8)	**0.57 (0.40, 0.81)**	**0.77 (0.66, 0.89)**
Drug Abuse ^e^						
None	6618	5659 (85.5)	845 (12.8)	114 (1.7)	1.00	1.00 ^h^
At least some ^f^	7084	6134 (86.5)	812 (11.5)	138 (2.0)	0.88 (0.68, 1.30)	1.13 (1.00, 1.25)
Missing	1546	1264 (81.8)	239 (15.5)	43 (2.8)	**0.61 (0.43, 0.87)**	**0.79 (0.68, 0.92)**
Criminal Risk rating ^g^						
Low	2876	2425 (84.3)	371 (12.9)	80 (2.8)	1.00	1.00 ^i^
Medium	6434	5500 (85.5)	831 (12.9)	103 (1.6)	**1.76 (1.31, 2.36)**	0.99 (0.87, 1.13)
High	5625	4877 (86.7)	644 (11.4)	104 (1.9)	**1.52 (1.13, 2.04)**	**0.86 (0.75, 0.99)**
Missing	313	255 (81.5)	50 (16.0)	8 (2.6)	1.09 (0.52, 2.29)	0.78 (0.56, 1.08)

^a^ Other races: collapsed together 28 different races, including Arab; Chinese; Japanese; Asian-West; British Isles, Caribbean, Euro-Northern

^b^ Other marital status includes: Single, Separated, Divorced, Widowed, Not Determined, Unknown, Missing

^c^ As defined by Problems Related to Drinking Scale (PRD)

^d^ As defined by Alcohol Dependence Scale (ADS)

^e^ As defined by the Drug Abuse Screening Test (DAST)

^f^ Combines together Individuals with Low, Moderate, Substantial and Severe history of drug abuse

^g^ As defined by Static Risk Factor Rating^34^

^h^ estimated for 13450 persons

^i^ estimated for 14648 persons

Table 2 (column 7) also presents the odds ratios for being matched deterministically compared with probabilistically. These odds did not vary significantly by age, marital status, or substance use histories. Those identifying as either black or ‘other’ races were less likely to be linked deterministically compared with those identifying as white. Having more aliases and having a higher criminal history risk rating was also associated with lower odds of deterministic linkage.

## Discussion

We linked records from almost 15,000 Ontario-based, federally incarcerated individuals to a province-wide health registry. This translated to 98% linkage rate and most records (86%) were linked deterministically. Our observed deterministic match rate is comparable to rates reported in other Canadian studies using the same data fields[24].

While the high linkage rate for Ontario-based persons is reassuring, we found that women, minority populations, and substance users were less likely to be linked to health records. Variations in linkage by participant characteristics are not surprising and have been reported elsewhere[31]. Reasons for lower linkage and the consequent underrepresentation of certain groups in the final database are not clear. A possible explanation for the lower linkage of women may relate to surname changes arising from marriage or divorce. We included a surname record for *all documented* aliases, but if different databases did not have the same surname on file, records could not have been linked. A possible explanation for the lower linkage rate of Indigenous Canadians may relate to jurisdictional differences in health care delivery. In Canada, the federal government is responsible for the delivery of some health care to Status Indians, including primary and emergent care on reserve. Although Status Indians are eligible for provincial health care, they may or may not be registered, i.e. in the RPDB. We acknowledge that systematic exclusions of sub-populations may bias estimates generated from these data[32–35]. However, our relatively large sample size means that we are more likely to conclude minor differences between groups are statistically significant, raising the question of how different truly are the 2% of men and 3% of women who were not linked? This will require attention as we move to using these data in future research.

We also found that certain groups were more likely to be linked, including those with a higher static risk rating. Although initially surprising, indicators of past criminal history contribute to the overall static risk rating and being involved with the criminal justice system could itself facilitate contact with health care. Individuals are routinely seen by a physician within a period of days to weeks of admission to a provincial correctional facility. Institutional staff will request health card numbers for anyone who is in provincial custody for long enough to be seen by a physician who does not already have a health card number. Accordingly, individuals with a higher risk rating may be more likely to be in the RPDB *because* of their criminal history.

We also observed a positive relationship between the number of aliases and data linkage. Aliases are ubiquitous in correctional populations: women incarcerated in British Columbia reported an average of 2.7 surnames in a 2005 study[36]. Similarly high numbers of aliases have been reported in incarcerated men and are in line with what we report herein[37]. Intuitively, it makes sense that those with more names have a higher likelihood of being linked to health data. The quandary lies in how to manage these individuals in linkage studies; specifically, how does one maximize rejection of false matches (i.e. specificity) and acceptance of true matches (i.e. sensitivity)? Some researchers have excluded individuals with aliases in excess of a specified threshold[36], although such an approach may decrease the linkage sensitivity and, as a result, misestimate the burden of health outcomes in incarcerated persons[22]. Future linkage studies in correctional populations should continue to explore the optimal approach to including those with several names.

We also compared records linked deterministically to those linked probabilistically. On most indicators, there were no systematic variations by linkage method. Because there is less certainty if probabilistically linked data are true matches, a conservative approach may be to remove these individuals from the linked dataset. Future studies using these data should conduct sensitivity analyses to fully understand the implications of including or excluding probabilistically linked records.

Our correctional data repository will enable the generation of much-needed knowledge of health pre and post incarceration. Because the OMS records were linked to a registry of all individuals eligible for health care in Ontario, this repository can be used to compare the health of incarcerated persons to the general population. We can also use these data to examine variations in health within specific sub-populations of interest (e.g. Aboriginal). In regards to the latter, the OMS contains a variety of standardized instruments that would not ordinarily be captured with administrative data (e.g., confounders, mediators).[16] These powerful data have the potential to contribute substantially to the field of health research in incarcerated persons.

The limitations of this study should be considered. The OMS contains records for all Canadians admitted to federal custody between 1998 and 2011, yet we only had access to Ontario health data. We have no way of knowing the true denominator for federal inmates eligible for health care in Ontario from these data. Because region of admission is often determined by the region the criminal offence was committed and where sentencing occurred, we restricted the majority of analyses to those admitted to an Ontario facility. The assumption being if region of crime and region of residence are interchangeable, those admitted to an Ontario facility should be in the RPDB. It is unclear if the 295 unlinked Ontario-based records were true missed linkages or if they belonged to people who resided outside of Ontario and could not have been linked. On a related note, 20% of inmates admitted to a federal facility outside of Ontario were linked to the RPDB, suggesting some had residence in Ontario over their lifecourse, and perhaps prior to incarceration.

## Conclusion

Through data linkage, we created a powerful platform to enable future research on the health of Canadian individuals who were incarcerated, pre- and post-incarceration. Our repository of almost 15,000 individuals federally incarcerated in Ontario includes demographic measures and validated instruments on substance use. Linkage to the RPDB, the registry of persons eligible for health care in Ontario, will enable the exploration of the health utilization and status of individuals using a variety of different indicators derived from physician billings, hospital separations, and emergent care databases. Although linkage rates in Ontario were high (98%), some populations were proportionately under-represented, like women and minorities.

## Supporting Information

S1 File Grey area resolution rules. (DOCX)

## References

[1] Correctional Services Program. Adult correctional statistics in Canada, 2013/2014. Juristat2015.

[2] Hawton K, Linsell L, Adeniji T, Sariaslan A, Fazel S. Self-harm in prisons in England and Wales: an epidemiological study of prevalence, risk factors, clustering, and subsequent suicide. The Lancet. 2014;383(9923):1147–54. 10.1016/S0140-6736(13)62118-2 PMC3978651 24351319

[3] Spaulding AC, Seals RM, Page MJ, Brzozowski AK, Rhodes W, Hammett TM. HIV/AIDS among inmates of and releasees from US correctional facilities, 2006: declining share of epidemic but persistent public health opportunity. PLoS One. 2009;4(11):e7558 10.1371/journal.pone.0007558 19907649 PMC2771281

[4] Baussano I, Williams BG, Nunn P, Beggiato M, Fedeli U, Scano F. Tuberculosis incidence in prisons: a systematic review. PLoS Medicine. 2010;7(12):e1000381 10.1371/journal.pmed.1000381 21203587 PMC3006353

[5] Fazel S, Bains P, Doll H. Substance abuse and dependence in prisoners: a systematic review. Addiction. 2006;101(2):181–91. 16445547 10.1111/j.1360-0443.2006.01316.x

[6] Fazel S, Danesh J. Serious mental disorder in 23 000 prisoners: a systematic review of 62 surveys. The Lancet. 2002;359(9306):545–50. 10.1016/S0140-6736(02)07740-1 11867106

[7] Kouyoumdjian FG, Schuler A, Hwang SW, Matheson FI. Research on the health of people who experience detention or incarceration in Canada: a scoping review. BMC Public Health. 2015;15:419 Epub 2015/05/07. 10.1186/s12889-015-1758-6 ; PubMed Central PMCID: PMCPmc4443600. 25943182 PMC4443600

[8] Wilper AP, Woolhandler S, Boyd JW, Lasser KE, McCormick D, Bor DH, et al The health and health care of US prisoners: results of a nationwide survey. American Journal of Public Health. 2009;99(4):666–72. 10.2105/AJPH.2008.144279 19150898 PMC2661478

[9] Kinner SA, Jenkinson R, Gouillou M, Milloy M-J. High-risk drug-use practices among a large sample of Australian prisoners. Drug and Alcohol Dependence. 2012;126(1):156–60. 22658284 10.1016/j.drugalcdep.2012.05.008

[10] Merrall EL, Kariminia A, Binswanger IA, Hobbs MS, Farrell M, Marsden J, et al Meta‐analysis of drug‐related deaths soon after release from prison. Addiction. 2010;105(9):1545–54. 10.1111/j.1360-0443.2010.02990.x 20579009 PMC2955973

[11] Zlodre J, Fazel S. All-cause and external mortality in released prisoners: systematic review and meta-analysis. American Journal of Public Health. 2012;102(12):e67–e75. 10.2105/AJPH.2012.300764 23078476 PMC3519300

[12] Ahalt C, Binswanger IA, Steinman M, Tulsky J, Williams BA. Confined to ignorance: the absence of prisoner information from nationally representative health data sets. Journal of General Internal Medicine. 2012;27(2):160–6. 10.1007/s11606-011-1858-7 21922160 PMC3270223

[13] Ahalt C, Trestman RL, Rich JD, Greifinger RB, Williams BA. Paying the Price: The Pressing Need for Quality, Cost, and Outcomes Data to Improve Correctional Health Care for Older Prisoners. Journal of the American Geriatrics Society. 2013;61(11):2013–9. 10.1111/jgs.12510 24219203 PMC3984258

[14] Wang EA, Wildeman C. Studying health disparities by including incarcerated and formerly incarcerated individuals. JAMA. 2011;305(16):1708–9. 10.1001/jama.2011.532 21521854 PMC5476220

[15] Binswanger IA, Redmond N, Steiner JF, Hicks LS. Health Disparities and the Criminal Justice System: An Agenda for Further Research and Action. Journal of Urban Health: Bulletin of the New York Academy of Medicine. 2012;89(1):98–107. 10.1007/s11524-011-9614-1 . PMC3284594 21915745

[16] Jutte DP, Roos LL, Brownell MD. Administrative Record Linkage as a Tool for Public Health Research. Annual Review of Public Health. 2011;32(1):91–108. 10.1146/annurev-publhealth-031210-100700 . 21219160

[17] Kinner SA, Forsyth S, Williams G. Systematic review of record linkage studies of mortality in ex-prisoners: why (good) methods matter. Addiction. 2013;108(1):38–49. 10.1111/add.12010 23163705

[18] Andrews DA, Bonta J, Hoge RD. Classification for effective rehabilitation: Rediscovering psychology. Criminal Justice and Behavior. 1990:19–52.

[19] Bonta J, Andrews D. Risk-Need-Responsivity Model for Offender Assessment and Rehabilitation 2007–06. Ottawa: Public Safety Canada; 2007.

[20] Skinner HA. The drug abuse screening test. Addictive behaviors. 1982;7(4):363–71. 7183189 10.1016/0306-4603(82)90005-3

[21] Iron K, Zagorski B, Sykora K, Manuel DG. Living and dying in Ontario: an opportunity for improved health information Toronto: Institute for Clinical Evaluative Sciences, 2008.

[22] Larney S, Burns L. Evaluating health outcomes of criminal justice populations using record linkage: the importance of aliases. Evaluation Review. 2011;35(2):118–28. 10.1177/0193841X11401695 21398273

[23] Jaro MA. Probabilistic linkage of large public health data files. Statistics in Medicine. 1995;14(5–7):491–8. 10.1002/sim.4780140510 7792443

[24] Li B, Quan H, Fong A, Lu M. Assessing record linkage between health care and Vital Statistics databases using deterministic methods. BMC health services research. 2006;6:48 10.1186/1472-6963-6-48 . 16597337 PMC1534029

[25] Skinner HA, Allen BA. Alcohol dependence syndrome: measurement and validation. Journal of abnormal psychology. 1982;91(3):199 7096790 10.1037//0021-843x.91.3.199

[26] Selzer M. The Michigan alcoholism screening test: the quest for a new diagnostic instrument. American journal of psychiatry. 1971;127(12):1653–8. 5565851 10.1176/ajp.127.12.1653

[27] Hodgins DC, Lightfoot LO. The Use of the Alcohol Dependence Scale with Incarcerated Male Offenders. International Journal of Offender Therapy and Comparative Criminology. 1989;33(1):59–67. 10.1177/0306624x8903300107

[28] Kunic D, Grant B. The computerized assessment of substance abuse (CASA): results from the demontration project Ottawa: Correctional Service of Canada, 2006.

[29] Correctional Service Canada. Commissioner's Directive: Correctional planning and criminal profile. Number 705–6 2014.

[30] Motiuk L. Classification for correctional programming: The Offender Intake Assessment (OIA) process. Forum on Corrections Research. 1997;19(1):18–22.

[31] Bohensky MA, Jolley D, Sundararajan V, Evans S, Pilcher DV, Scott I, et al Data linkage: a powerful research tool with potential problems. BMC health services research. 2010;10:346 Epub 2010/12/24. 10.1186/1472-6963-10-346 ; PubMed Central PMCID: PMCPmc3271236. 21176171 PMC3271236

[32] Schmidlin K, Clough-Gorr KM, Spoerri A, Egger M, Zwahlen M. Impact of unlinked deaths and coding changes on mortality trends in the Swiss National Cohort. BMC medical informatics and decision making. 2013;13:1 Epub 2013/01/08. 10.1186/1472-6947-13-1 ; PubMed Central PMCID: PMCPmc3547805. 23289362 PMC3547805

[33] Harron K, Wade A, Gilbert R, Muller-Pebody B, Goldstein H. Evaluating bias due to data linkage error in electronic healthcare records. BMC Med Res Methodol. 2014;14:36 Epub 2014/03/07. 10.1186/1471-2288-14-36 ; PubMed Central PMCID: PMCPmc4015706. 24597489 PMC4015706

[34] Draper GK, Somerford PJ, Pilkington AS, Thompson SC. What is the impact of missing Indigenous status on mortality estimates? An assessment using record linkage in Western Australia. Aust N Z J Public Health. 2009;33(4):325–31. 10.1111/j.1753-6405.2009.00403.x . 19689592

[35] O'Reilly D, Rosato M, Connolly S. Unlinked vital events in census-based longitudinal studies can bias subsequent analysis. Journal of Clinical Epidemiology. 2008;61(4):380–5. 10.1016/j.jclinepi.2007.05.012 18313563

[36] Martin RE, Hislop TG, Grams GD, Moravan V, Calam B. Beware of multiple names in database linkage research: prevalence of aliases in female prison population. British Medical Journal. 2005;331(7512):335–6. . 16081448 10.1136/bmj.331.7512.335 PMC1183136

[37] Harry B. Diagnostic study of the criminal alias. J Forensic Sciences. 1986;31(1023):8.

