# Supplementary material for: Creating a Powerful Platform to Explore Health in a Correctional Population: A Record Linkage Study
Source: PLoS One. 2016 Aug 17;11(8):e0161173. doi: 10.1371/journal.pone.0161173 (PMC4988706; doi:10.1371/journal.pone.0161173)
Supplement: S1 File — (DOCX) [file pone.0161173.s001.docx]

###### S1: Grey area resolution rules

1. AGREEMENT ON SURNAME, NAME1, DATE OF BIRTH --> **MATCH**

2. AGREEMENT ON SURNAME, NAME1, TRANSPOSITION OF BIRTH MONTH AND DAY --> **MATCH**

3. AGREEMENT ON SURNAME, DATE OF BIRTH, TRANSPOSITION OF NAME1 AND NAME2 (if available) --> **MATCH**

4. AGREEMENT ON SURNAME, DATE OF BIRTH, NAME1 NICKNAME VARIANT --> **MATCH**

5. AGREEMENT ON SURNAME, NAME 1 INITIAL, DATE OF BIRTH, DATE OF DEATH --> **MATCH**

6. AGREEMENT ON UNCOMMON SURNAME, PARTIAL NAME 1, DATE OF BIRTH --> **MATCH**

7. AGREEMENT ON SURNAME, NAME1 INITIAL, DISAGREEMENT ON DATE OF BIRTH --> **NO MATCH**

8 AGREEMENT ON SURNAME, NAME1 BUT DISAGREEMENT ON NAME2 INITIAL (where available), DATE OF BIRTH --> **NO MATCH**

9. AGREEMENT ON SURNAME, NAME2 INITIAL BUT FULL DISAGREEMENT ON NAME1, DATE OF BIRTH --> **NO MATCH**

10. AGREEMENT ON SURNAME, DATE OF BIRTH, NAME1 ABSENT --> **NO MATCH**

##

## Grey Area Resolution rules: Rationale

Definitions:

“Minor” Disagreement occurs where:

a) Slight discrepancy occurs in the reported surname or given names

b) Only 1 birth date component disagrees (i.e., birth year / month / day).

“Major” disagreement:

1. Surname or given names disagree completely

Note: nicknames should not be considered a major disagreement

e.g., Margaret vs. Peggy; Nicholas vs. Nick; Elizabeth vs. Betty;

b) Two birth date components are in disagreement (e.g., month and day are not the same)

NB: transpositions between month and day can occur; this type of discrepancy should be considered a “minor” disagreement.

1. Death dates differs significantly

General “Rules of Thumb” for Adjudicating / Resolving Clerical Pairs:

- **Accept** pair if:

1. 1 or 2 minor disagreements

- **REJECT** pair if:
  - 1 major disagreement in surname + 1 minor disagreement in DOB
  - 2 or more major disagreements

**General motto: *“IF IN DOUBT, THROW IT OUT!”***
